# [Retracted] RNAi‑mediated EZH2 depletion decreases MDR1 expression and sensitizes multidrug‑resistant hepatocellular carcinoma cells to chemotherapy

**DOI:** 10.3892/or.2024.8809

**Published:** 2024-09-09

**Authors:** Bo Tang, Yi Zhang, Rui Liang, Zhenming Gao, Deguang Sun, Liming Wang

Oncol Rep 29: 1037–1042, 2013; DOI: 10.3892/or.2013.2222

Following the publication of this paper, it was drawn to the Editor's attention by a concerned reader that the control western blotting data featured in Fig. 2C on p. 1039 and the cell cycle distribution images shown in Fig. 6A on p. 1041 were strikingly similar to data that had appeared in a pair of other articles written by different authors at different research institutes, one of which had already been submitted for publication when this article was received at *Oncology Reports*, the other of which was received some time afterwards, but which has subsequently been retracted. Owing to the fact that the abovementioned data had already been submitted for publication prior to its submission to *Oncology Reports*, the Editor has decided that this paper should be retracted from the Journal. The authors were asked for an explanation to account for these concerns, but the Editorial Office did not receive a reply. The Editor apologizes to the readership for any inconvenience caused.

